# Uveal Melanoma Ground Truth Labeling in Machine Learning

**DOI:** 10.3390/cancers18091357

**Published:** 2026-04-24

**Authors:** Emily Kao, Sanjay Ganesh, William F. Chadwick, Reem Alahmadi, Xincheng Yao, Michael J. Heiferman

**Affiliations:** 1 Department of Ophthalmology and Visual Sciences, University of Illinois Chicago, Chicago, IL 60607, USA; 2 Department of Ophthalmology, Boston Children’s Hospital, Boston, MA 02115, USA; 3 Department of Biomedical Engineering, University of Illinois Chicago, Chicago, IL 60607, USA

**Keywords:** uveal melanoma, artificial intelligence, ground truth, ocular oncology

## Abstract

Background/Objectives: Uveal melanoma (UM) is the most common primary intraocular malignant tumor among adults and has a high risk of metastasis. Recently, artificial intelligence (AI) tools have been developed to support the management of UM across different clinical tasks. The definition of ground truth, the reference standard that models use in training and development, greatly influences the performance and clinical relevance of the models. Currently, there is limited consensus regarding which ground truth methods are most appropriate for each clinical application. This review aims to evaluate the advantages and limitations of available ground truth options in UM and proposes task-specific recommendations based on clinical utility, feasibility, and cost. Methods: A narrative review of the existing literature was conducted to identify and evaluate commonly used ground truth methods for UM AI applications based on factors such as time, cost, invasiveness, and required level of expertise. Results: Each ground truth method offers distinct benefits and drawbacks in relation to biological precision, invasiveness, availability, cost, and turnaround time. No single ground truth is universally optimal across all applications. Instead, the ideal choice depends on the intended clinical task, and practical alternatives exist to mitigate the constraints that result from limited time and institutional resources. Conclusions: The selection of ground truth for AI models in UM should be chosen based on the specific clinical task to balance predictive relevance with feasibility of implementation. The adoption of task-specific ground truth standards may improve the development of clinically meaningful AI tools and facilitate their integration into real-world practice.

## Simple Summary

Artificial intelligence tools are increasingly being developed to support the management of patients with uveal melanoma, the most common intraocular malignancy, across clinical tasks such as screening, diagnosis, prognostication, and treatment planning. The accuracy of these tools depends on the definition of ground truth, which is the reference standard that models use in training and algorithm development. There is currently a lack of consensus on which ground truth methods are most appropriate for each clinical application. This review evaluates the benefits and drawbacks of ground truth methods, including clinical diagnosis, genetic profiling, histopathology, and long-term outcomes, and examines how well they align with real-world clinical goals, costs, and feasibility. Ultimately, this review proposes task-specific ground truth choices as well as practical alternatives, aiming to guide the development of tools that are more clinically relevant, cost-effective, and better integrated into patient care settings.

## 1. Introduction

Uveal melanoma (UM) is the most common primary intraocular malignant tumor among adults [1]. It is frequently associated with vision loss and a high risk of metastasis to the liver. Once metastatic disease develops, the prognosis is poor, contributing to an estimated mortality of 50% within 10 years of the initial primary tumor diagnosis [2,3]. Early detection and intervention are therefore critical for improving patient outcomes, as current treatment is effective in controlling small, nonmetastatic tumors [4,5]. However, UM shares a similar clinical presentation with indeterminate or benign melanocytic choroidal tumors such as nevi, which makes early and accurate differentiation difficult [6].

Recently, artificial intelligence (AI), and machine learning (ML) in particular, have emerged as valuable tools for automating and enhancing clinical tasks such as image classification, semantic segmentation, and clinical prediction [7,8,9,10]. Previous studies have demonstrated the utility of AI in UM-specific applications, from using multi-modal imaging to predict malignant transformation to directly projecting survival status from cytopathology slides [11,12,13]. AI can enable complex multivariate analyses of imaging, clinical, and molecular data that reveal patterns beyond the scope of human decision-making, which cognitive research shows considers only up to four variables at a time [14]. Additionally, AI automation has the potential to improve current clinical workflows in screening, triaging, and longitudinal monitoring. Given the limited worldwide availability of ocular oncologists, streamlining tasks may help facilitate timely treatment by increasing volume and enabling less specialized practitioners to take over certain points of care [15]. Most recently, advances in extracting features and optimization of model performance have begun to bring the current state-of-the-art closer to clinical deployment [16,17].

Regardless of the field or application of individual AI models, common practice uses “ground truth” datasets as the basis for metric analysis and for training algorithms against external standards [18]. In other words, ground truths serve as definitive classifications that, in clinical settings, can take the form of retrospective clinical data labels, manual annotation of radiological or histopathological images, consensus definitions, etc. [19,20,21]. However, while ground truths are meant to mimic neutral reality and operate under the implicit assumption of perfect accuracy, they inevitably reflect the collection and processing choices of the associated ground truth dataset, which can significantly affect model quality [22,23]. As a result, ground truth datasets are best conceptualized as a reference standard, serving as a comparator against which model performance can be evaluated rather than an objective truth. The choice of ground truth is therefore a crucial decision that can determine model performance and clinical utility [24].

Evaluation of ground truth selection has been conducted in other fields to optimize predictive power. For example, one study in pediatric trauma evaluated the performance of three different ground truth labels for patient triage. Each method had its tradeoffs, with one under-triaging patients with stab injuries and another over-triaging patients requiring airway management. However, since sensitivity and patient safety are most important for the given task, a combination of the two scoring systems was determined to be the best fit [25].

Thus, the choice of ground truth must be carefully defined in accordance with the clinical task the ML model aims to support to reduce subjectivity and achieve the clinical relevance necessary for safe and effective implementation. In UM specifically, AI has the potential to enhance diagnostic and prognostic accuracy, automate triage, and aid in treatment decisions [26]. However, the relative rarity of UM makes it difficult to obtain some ground truth choices, and other, more readily available choices may be high-cost, require more computing power, or take significant time to acquire. Accordingly, balancing data needs, accessibility, and cost with the degree of clinical reflection is especially important in UM AI applications to preserve both validity and feasibility.

**Figure 1:**
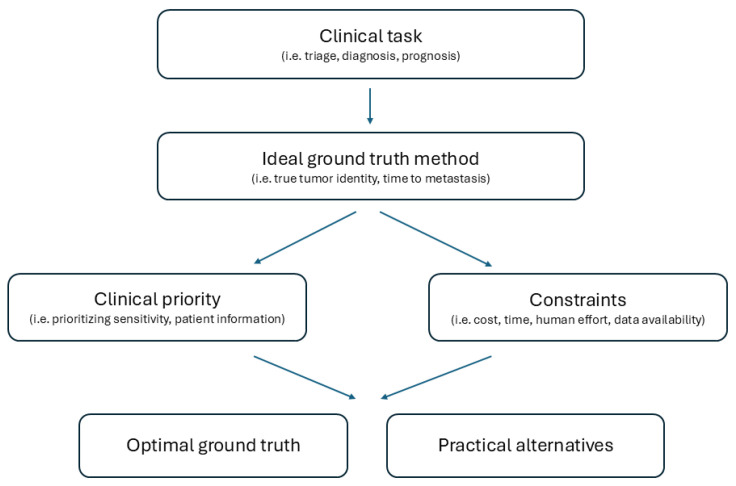
Task-based framework for ground truth selection in UM AI.

While prior reviews have examined AI applications in UM, this review will specifically analyze the benefits and drawbacks of possible ground truth options as a central determinant of model performance and clinical relevance, ultimately proposing ground truth choices for each particular task. It will also explore practical alternatives and key limitations for each choice, with an emphasis on their cost-effectiveness and integration into real practice, and conclude with potential methods and areas for improving current practices. This framework is summarized visually in Figure 1.

## 2. Methods

A literature search was performed using databases including PubMed, Google Scholar, and Scopus. Keyword search terms included combinations of “uveal melanoma,” “artificial intelligence,” “ground truth,” “diagnosis,” “prognosis,” and “prediction.” Articles were selected based on relevance to ground truth methodology and clinical applications of AI in UM or related oncologic contexts. Given the narrative nature of this review, formal inclusion and exclusion criteria were not strictly predefined. The publication timeframe of literature considered ranged from 1990 to the present, with emphasis placed on studies published within the past 10 years.

## 3. Overview of UM Ground Truth Methods

Multiple ground truth methods have been employed in UM research, ranging from genetic profiling to long-term patient outcomes, each reflecting different biological, clinical, or outcome-based definitions of disease, and therefore suited to different clinical tasks.

### 3.1. Clinical Examination

Clinical examination serves as a commonly used ground truth for UM AI models due to its immediacy and non-invasiveness [19]. Upon presentation, this approach evaluates established clinical characteristics such as the presence of subretinal fluid and orange pigment, as well as observations of tumor thickness, lesion diameter, number, location, and growth characteristics [27].

Two main staging systems have been developed based on the clinical approach to indicate risk of metastasis. The Collaborative Ocular Melanoma Study (COMS) stratifies disease based on tumor height and width, with studies reporting misprediction rates as low as 0.48% [28]. The American Joint Committee on Cancer (AJCC) also evaluates evidence of regional or distant spread in addition to tumor dimensions, with the rate of metastasis or death increasing three- and ten-fold across stages one through three, respectively [29]. While these frameworks are valuable for clinical decision-making, they rely partly on tumor size thresholds, which in isolation may fail due to considerable overlap between benign lesions such as large nevi and small malignant melanomas [30]. As a result, this could limit their utility as discrete ground truths for AI classification tasks.

While the accuracy of clinical diagnosis has continued to be optimized, inherent influences from clinicians’ experience and institutional practice patterns may introduce subjectivity and inter-observer variability when used as ground truth. For example, measurement of tumor dimensions such as thickness and largest basal diameter from ultrasound is highly dependent on operator experience and equipment type, therefore introducing an element of variation in measurement that makes objectivity difficult [31]. Additionally, lesions may be classified in the clinic as indeterminate melanocytic choroidal tumors (IMCT), which is an uncertain diagnosis when reviewed retrospectively without adequate patient follow-up [32]. In such cases, clinical classification may also be influenced by management decisions made at the time of presentation. For example, if patients elect to undergo treatment, their lesion is more likely to be diagnosed as melanoma, while a mutual decision to manage and observe is more likely to be classified as nevi. As a result, this introduces a risk of circularity that can bias ground truth assignments [32]. Therefore, although clinical diagnosis remains highly accurate in expert settings, the absence of standardized definitions distinguishing nevi from small UM may pose challenges for model development and validation when used as a standalone ground truth, as inconsistency in interpretations may introduce label noise that can significantly degrade predictive performance [14].

### 3.2. Histopathology

Histopathologic assessment is another traditional ground truth method in UM, providing detailed characterization of tumor morphology that may inform both classification and prognostication in a more objective manner than clinical evaluation [33]. Microscopic evaluation of histopathology slides enables differentiation between spindle, epithelioid, and mixed cell types, with epithelioid morphology associated with more aggressive clinical behavior [33]. Additional features, including mitotic activity, tumor-infiltrating lymphocytes, and extravascular matrix patterns, have also been correlated with metastatic risk and overall prognosis [34]. Previous studies have found the accuracy of histopathologic confirmation of diagnoses to be as high as 99.7% [33].

While histopathological features are the traditional standard for diagnosing and prognosticating UM, their utilization may fail in the case of small lesions where tissue sampling is limited [35]. Additionally, patients and providers may be hesitant to biopsy smaller or lower-risk lesions due to the potential for procedural risks such as hemorrhage, retinal detachment, or cataract, which may limit representative sampling [36]. In the same vein, most studies on histopathologic characteristics have been performed on samples from enucleated eyes, which may introduce selection bias and limit model generalizability to earlier-stage disease [33]. The use of histopathology as a ground truth may also be constrained by the availability of experienced ocular pathologists, introducing potential variability in interpretation across institutions, restricting its applicability as a universal ground truth [37].

### 3.3. Genetic Profiling

Genetic profiling has more recently emerged as an important ground truth method in UM, with gene expression profiling (GEP), in particular, being validated for risk stratification and prognostication [38]. The process involves obtaining a tumor biopsy specimen and completing molecular analysis, creating a 15-gene expression profile that predicts 5-year metastatic risk [39]. Patients are stratified into two classes based on previously determined associations with different prognoses; for example, class 2 genetic profiles are often associated with mutations in the BRCA1-associated protein 1 (BAP1) tumor suppressor gene on chromosome 3, and expression of Preferentially Expressed Antigen in Melanoma (PRAME), which are strongly linked to increased risk of metastasis and poor prognosis [40]. Several studies have shown high accuracy and technical reliability, with one demonstrating successful classification of 97.2% of cases, surpassing the ability of anatomical categorization [40]. In addition to GEP, common alterations in the genome associated with UM include monoallelic loss of chromosome 3, gain of the long arm of chromosome 8, loss of the short arm of chromosome 6, and mutations in EIF1AX and SF3B1, which may serve as important biomarkers for model training [41].

Molecular approaches that utilize techniques such as next-generation sequencing (NGS) and multiplex fluorescence in situ hybridization (MFISH) may be preferred due to their ability to provide individualized insight into intrinsic tumor biology, their resistance to interpretive bias, and their label stability [32]. However, there is still ongoing debate regarding whether molecular classification offers enough improved prognostic accuracy compared to simpler, non-invasive clinical indicators such as tumor size to compensate for increased costs [42]. Additionally, accessibility of GEP varies by institution, which may introduce data source considerations that run the risk of perpetuating substandard predictions for underrepresented groups [43,44]. Overall, GEP represents a promising ground truth method that can also be utilized in combination with other methods and may continue to evolve as larger and more diverse prospective datasets are acquired [45].

### 3.4. Risk Factor-Based Scoring Tools

Risk factor-based scoring systems such as MOLES (Mushroom shape, Orange pigment, Large size, Enlarging tumor, Subretinal fluid) and TFSOM (Thickness, subretinal Fluid, Symptoms, Orange pigment, Margin), which represent the most well-known predictive indicators of malignancy, have been developed to support triage for melanocytic choroidal lesions [3,46]. For example, in non-specialist settings where advanced imaging, biopsy, or molecular testing may not be readily available, MOLES scores can be used to categorize tumors from “common nevus” to “probable melanoma”, informing treatment decisions and urgency of referrals [47]. Several studies have validated MOLES to have specificity and sensitivity values of up to 96% and 100%, respectively [48]. However, reliance on probabilistic risk assessment rather than discrete diagnostic classification may limit their precision when used as standalone labels in model development for treatment decisions [46]. Additionally, long-term outcomes may not be available for prediction confirmation, as treatment is administered when TFSOM or MOLES scores indicate malignancy.

### 3.5. Manual Image Annotation

Manual annotation of ophthalmic imaging by expert clinicians is another, albeit more limited in scope, ground truth for UM AI models. It is mainly relevant to segmentation rather than classification tasks, in which AI aids in identifying and evaluating areas of interest within images [19]. In this approach, ocular oncologists delineate tumor boundaries or identify relevant features on multi-modal imaging to generate labeled datasets, which has been similarly implemented in other fields such as radiology [49]. This method enables the incorporation of specialist knowledge directly into model training and may closely reflect real-world clinical interpretation; however, it is inherently time- and labor-intensive, requiring significant expert input [49]. Additionally, it may be subject to inter-observer variability, which can affect scalability when used as a reference standard.

### 3.6. Prospective Monitoring

Long-term clinical outcomes, including metastasis and disease-specific survival, represent the most clinically relevant ground truths for many ML models of UM, as they directly reflect patient-centered endpoints, and predicting survival outcomes is a central objective of numerous clinical tasks [26]. The use of such endpoints as ground truth may present significant practical challenges, though, as outcome data are inherently time-consuming to obtain prospectively and may only become available months to years following diagnosis [50]. Additionally, tumor labels may not always remain stable when assigned after long periods of time, unlike characteristics such as genetic markers, as malignant transformation can occur at any point after initial presentation [2]. Furthermore, the collection of sufficiently large prospective datasets in this rare disease is resource-intensive and costly, similarly demonstrated in other oncological studies, limiting the current feasibility of training robust models based on long-term follow-up alone [51].

Survival studies in particular are prone to bias, as competing risks of death may confound statistical analyses. One alternative to evaluate treatment efficacy that can also shorten follow-up periods is by tracking outcomes other than survival, such as the disappearance of tumor DNA or circulating tumor cells, that serve as real-time indicators for monitoring remission and disease progression [52].

Prospective monitoring as a whole, however, is crucial to the iterative nature of ML model development, allowing for those trained on available ground truths to be evaluated against true outcomes and subsequently refined to improve predictive performance. This feedback loop may help mitigate limitations associated with proxy labels or dataset shifts, especially in settings where the most ideal ground truths are not immediately available [53]. Models may then converge toward more clinically meaningful representations of disease behavior over time.

Collectively, the strengths and limitations of these ground truth methods highlight that no single approach is universally optimal across all clinical applications in UM (Table 1). Rather, the appropriateness of a given ground truth is inherently dependent on the demands of the specific task being addressed in concurrence with considerations of resource availability, human effort, cost, and time.

## 4. Ideal Ground Truths by Clinical Task

A task- and resource-oriented framework for ground truth selection is therefore necessary to ensure that AI models in UM are both clinically relevant and operationally feasible. Accordingly, the following section outlines task-specific recommendations for ground truth selection in the development and implementation of AI models in UM.

### 4.1. Automated Triage

Given the limited availability of specialized ocular oncologists, triage of indeterminate melanocytic choroidal lesions represents a high-impact, resource-sensitive decision point where AI may provide substantial clinical and operational benefit [26]. Clinical diagnosis seems to represent the most appropriate ground truth, as it closely reflects real-world outcomes following referral to an ocular oncologist and is both accurate and scalable [28]. However, if the resulting clinical diagnosis is unavailable, such as in cases of IMCTs, structured risk-based scoring systems such as MOLES offer a practical, more stratified alternative. Ground truths would then more closely simulate triage decision-making in non-specialist settings. While less biologically precise than expert clinical assessment, this approach aligns with the primary objective of preliminary screening due to its emphasis on maintaining high sensitivity in identifying lesions that warrant referral, to reduce the risk of delayed diagnosis in patients with suspected UM [46]. In one recent study, validation of the MOLES criteria demonstrated increased sensitivity from previously reported 97.9% to 100%, underscoring the prioritization of patient safety for the purposes of triage tasks [54].

### 4.2. Initial Diagnosis

In clinical practice, AI-based initial diagnostic tools are being developed for UM that would be incorporated to assist and support early clinical decision-making by distinguishing melanocytic lesions upon presentation. An ideal ground truth would be derived from conducting prospective studies that evaluate variation in long-term clinical outcomes based on presenting clinical features and imaging findings at the time of diagnosis. Lesions would then be classified according to their likelihood of contributing to downstream outcomes in addition to histologic or molecular confirmation of malignancy, aligning diagnostic labeling with clinically meaningful endpoints. One study, the Liverpool Uveal Melanoma Prognosticator Online (LUMPO), has used these techniques to identify high-risk UM patients at a large multicenter scale, with a follow-up time of more than 20 years. Using clinical data only, this tool demonstrated an index of discrimination of around 0.75 [55]. However, relatively few other AI-based diagnostic models have retroactively integrated these correlations into a ground truth dataset, instead relying on current clinical diagnosis as a surrogate [56,57]. This may be due to the substantial time, cost, and logistical demands associated with prospectively collecting longitudinal outcome data for such a rare disease, as well as the potential bias introduced by the impact of other comorbidities on survival and the tendency to treat suspected melanomas without delay upon presentation.

More practical alternatives for ground truths that are less resource-intense than long-term follow-up may be any choice or combination of more traditionally used methods, such as a retrospective clinical diagnosis, which is true to the decision made at presentation, or tissue analysis methods like histopathology and genetic profiling. Histopathology serves as the traditional standard for tissue-level confirmation but may be clinically misaligned, since biopsy is not always performed at initial presentation and can be subject to bias when data is collected from institutions that tend to only perform biopsies on cases severe enough to warrant enucleation [33]. While genetic methods are intended to be less biased by subjective interpretation, there are still gaps in the current understanding of the mutational profile of melanomas. For example, Class 2 GEP has also reported associations with non-melanocytic uveal metastases from other organs, and some studies show discordant classifications when samples are biopsied at different sites [58]. Overall, both methods are available for ground truth data collection within days to weeks of clinical presentation, making them more readily available and reflective of real-world diagnostic workflows, but they may fail to characterize the relationship between diagnosis and clinical impact.

### 4.3. Management Decision

For AI applications intended to support management decision-making in UM, long-term clinical outcomes are again the most clinically meaningful and ideal ground truths. Similar to initial diagnostic tasks, several previous studies have investigated long-term clinical outcomes of treatments such as plaque brachytherapy and proton-beam radiation [59,60]. This is particularly relevant when deciding whether to treat or observe IMCTs with similar clinical features and UM with genetically low-risk profiles. For example, studies like those associated with LUMPO have used long-term follow-up to show that deferring treatment of IMCTs until growth is observed is associated with only minimal risk of metastatic death [61]. Although randomized controlled trials (RCTs) remain the gold standard for evaluating management strategies, the time, cost, and logistical complexity of conducting such trials across the wide range of tumor sizes, clinical features, and GEPs make them largely impractical in this setting. As a more feasible alternative, consensus treatment recommendations via evaluation by a multidisciplinary tumor board may be a practical intermediate ground truth, reflecting expert-informed management strategies that aim to optimize long-term outcomes. This would more closely align with ideal treatment based on the individual, which can incorporate other factors such as patient or surgeon preference, and may be less noisy than retrospective labels. The creation of these ground truth datasets would be similar to consensus recommendations like those devised by the National Comprehensive Cancer Network (NCCN) but would be able to incorporate more specific aspects of clinical presentation and multimodal imaging [62].

In resource-constrained settings where the number of available ocular oncologists may limit the attainability of consensus-based data, the treatment actually administered at the time of presentation may be retrospectively collected as an even more pragmatic alternative ground truth. Even in some centers, electronic medical records have been configured to prospectively collect information on treatment patterns [63]. While this approach also incorporates patient preference, institutional resources, and physician experience, it may also limit the ability of models to distinguish optimal from feasible management strategies, as it lacks consideration for treatment plans not chosen due to these constraints [64].

### 4.4. Radiation Treatment Planning

Radiation treatment planning represents a technically focused clinical task in UM, where AI may augment precision in the delineation of tumor boundaries for localized therapy [26]. In this setting, expert manual annotation performed by ocular oncologists and subsequent treatment planning by radiation oncologists represent the ideal ground truth for model training, as it provides reference standards for outlining tumor boundaries and optimizing plaque placement for dose distribution [19].

Manual annotation by experts may also be time-consuming and resource-limited. An emerging practical alternative for ground truth choice is the use of unsupervised or partially supervised modeling approaches that may reduce reliance on manually labeled ground truths by leveraging similarity metrics, anatomical landmark detection, or automatically generated masks for contouring. Such techniques have been demonstrated in other oncologic contexts, including brain and lung tumor planning using CT and MRI imaging [65]. However, their application to ophthalmic imaging modalities like fundus photography or OCT remains underexplored, and the absence of current clinical validation in combination with opaque processes associated with self-supervision may present challenges for future implementation in ocular oncology workflows [14].

### 4.5. Long-Term Outcome Prediction

Long-term outcome prediction aims to estimate the percent chance that metastasis will occur rather than simply classify a lesion, as metastatic risk is variable in UM. As a result, it is most aligned with ground truth clinical endpoints obtained from prospective monitoring. These measures may enable features like time-to-event modeling, which have already been utilized and developed in the context of localized cutaneous melanoma [66]. Standalone outcome-based ground truths may be influenced by detection bias; however, earlier diagnosis or more intensive monitoring may become associated with improved observed outcomes independent of tumor biology [67]. Additionally, the use of overall survival as a proxy may be confounded by competing comorbidities unrelated to UM. Inconsistent labeling of the time to metastasis may also present challenges, as previous diagnoses of metastasis occurred upon detection in the liver rather than current metastatic diagnoses resulting from genetic typing. Given the implications for guiding decisions, models developed for this task may require a higher degree of evidentiary certainty. Therefore, studies validating greater predictive power for prospective monitoring compared to the current state of genetic profiling may be needed to assess future utility and implementation.

In current practice, composite multivariable analyses such as those performed by LUMPO that integrate clinical features, histopathology, and genetic profiling may serve as a more realistic surrogate for ground truth closer to the time of clinical presentation [12,29]. These classifications offer individualized outcome prediction and hold advantages over purely genetic methods due to easier communication and familiarity amongst non-specialists.

### 4.6. Patient Counseling

Prognostic counseling is a distinct application in which AI may assist in estimating individualized metastatic risk to inform life planning and support patient-centered decision-making [26]. Molecular profiling represents an appropriate ground truth, as it provides a degree of individualization that other methods currently lack, and higher-resolution insight into tumor biology and metastatic potential. Many patients elect to undergo prognostic testing despite the associated cost or invasiveness, as they derive value from information about their risk of metastasis regardless of their individual risk profile, reinforcing the importance of prognostic counseling in the context of long-term life planning [68].

Where the availability of GEP may vary globally, histopathologic features such as largest tumor diameter, cell type, and microvascular patterns may also contribute to prognostic assessment in a similar fashion. Current studies have already begun to use features extracted from whole-slide images to predict liver metastasis and stratify high vs. low-risk groups [69]. In some cases, histopathologic and genetic approaches may be combined to further enhance prognostic accuracy by capturing complementary biological and morphologic determinants of metastatic risk [69]. An AI model could improve personalized counseling by consistently integrating the predicted metastatic risk with the patient’s values, goals, and life expectancy to align surveillance intensity and management decisions with individualized life and care priorities.

As described in the previous section, the most appropriate form of ground truth in UM AI modeling is dependent on the clinical task, each with distinct data needs that ensure meaningful AI model development and validation (Table 2). The feasibility of implementing these ground truth standards in practice is ultimately constrained by real-world considerations such as data availability, computational resources, and the human capital required for acquisition, annotation, and integration within clinical workflows. Given these limitations, the following section will examine how current practices may be improved and outline future directions for optimizing ground truth selection and utilization in clinically deployable UM AI applications.

## 5. Areas for Improvement in Current Practice

While variation in ground truths makes several methods available for a given specific clinical function, current clinical and research practices in UM modeling may still be strengthened to eliminate some of the associated challenges. For example, broader adoption of standardized definitions and consensus-driven criteria established by multidisciplinary expert committees may improve consistency across both clinical practice and ML model development [57]. The implementation of universal size-based classification criteria would enable more consistent and interpretable stratification, which is especially important for computational models that rely on discrete labelling. Additionally, as UM models become more developed, multi-institutional standardized data collection becomes increasingly important to improve generalizability through the creation of high-quality, intentionally labelled datasets. Lastly, rather than relying on binary predictions of metastatic outcome, models should continue to focus on generating individualized probabilistic risk estimates that more accurately reflect clinical uncertainty and the actual mechanism of the disease [70]. From there, models could then strive to incorporate further holistic considerations like access to care, socioeconomic limitations, and geographic barriers to provide a more context-aware assessment of risk based on the healthcare system of the country of development [71]. Integrating these factors would allow for better support of patient counseling, allowing it to move beyond tumor biology alone and instead reflect the broader realities that shape diagnosis, treatment, and adherence.

## 6. Conclusions and Future Directions

Selecting an appropriate ground truth for AI in UM applications is not a one-size-fits-all decision, as it must be deliberately aligned with the intended clinical task and potential cost. As emphasized throughout this review, tasks such as initial diagnosis, radiation planning, and prognostic counseling each require fundamentally different ground truth characteristics for training and algorithm development to ensure the clinical meaning of the outputs. Objective ground truths based solely on histopathologic or genetic information may be preferred for classification-based tasks, yet they often lack holistic consideration of factors like socioeconomics that may also inform management decisions and impact survival, metastatic risk, or quality of life. Conversely, clinically anchored endpoints like disease-specific survival or time to metastasis are more reflective of real-world outcomes but require substantial longitudinal data and human effort to implement on larger scales.

Therefore, the feasibility of developing task-appropriate ground truths is constrained not only by conceptual considerations but also by the availability of high-quality datasets, institutional resources, and the degree of clinician involvement required for annotation and validation. Many current applications have relied on retrospective endpoints such as clinical diagnosis at presentation or treatment administered at the time of care due to practical limitations, which may introduce bias or reinforce existing assumptions embedded within standard practice [19]. Addressing these limitations will require greater emphasis on standardized definitions and outcome-oriented validation strategies that move beyond retrospective labeling and towards prospectively meaningful clinical endpoints [57].

Future directions for the application of AI in UM include developing models capable of integrating inputs ranging from imaging to molecular biomarkers to generate individualized probabilistic risk estimates while maintaining accurate and transparent ground truth datasets. Establishing task-based frameworks to guide ground truth selection may further facilitate the translation of ML tools into clinical environments by helping to balance ideal methodological rigor with pragmatic feasibility.

Ultimately, progression of AI models in UM and other related fields will depend on continued collaboration between clinicians, data scientists, and institutions to ensure that emerging algorithms are trained and validated with intentional ground truths that are aligned with the outcomes they are intended to influence. By aligning model development with clinically relevant ground truths, future ML systems may hold greater potential to ultimately predict disease behavior and meaningfully improve personalized care and long-term patient outcomes.

## Figures and Tables

**Table 1:** Summary of the ground truth methods available for AI applications in UM. + = low, ++ = moderate, +++ = high.

Ground Truth	Time	Cost	Invasiveness	Expertise	Subjectivity	Other Factors
Clinical examination	+	+	+	+++	+++	• IMCT labels leave true diagnosis un-known • May reflect clinician experience and management bias
Histopathology	++	++	+++	+++	++	• Traditional diagnostic reference standard • May not be possible for very small lesions
Genetic profiling	++	++	++	+	+	• Biologically specific and relatively objective • Not yet validated for diagnosis
Risk factor-based scoring tools (e.g., TFSOM, MOLES)	+	+	+	+	++	• High sensitivity • Facilitates standardized risk assessment
Manual annotation (e.g., chart/image review for diagnosis; drawing segmentation maps)	++	++	+	+++	++	• Essential for image segmentation-related tasks • Subject to inter-observer variability
Prospective monitoring (e.g., time to metastasis; clinical trial)	+++	+++	+	+	+	• Precise in relation to true, clinically relevant outcomes • Requires prolonged follow-up infrastructure

**Table 2:** Summary of ground truth choice recommendations by clinical task.

Clinical Task	Best-Aligned Ground Truth	Practical Alternatives
Automated triage	Specialist clinical diagnosis • Best reflects real-world referral triage after ophthalmic evaluation • Aligns model output with real-world referral workflows	Risk factor-based scoring tools (e.g., TFSOM, MOLES) • Useful in non-specialist settings • Prioritizes sensitivity and standardized risk stratification
Initial diagnosis	Prospective longitudinal outcome confirmation • Most biologically faithful method for distinguishing melanoma from indeterminate lesions • Reduces reliance on presentation-time assumptions	Specialist clinical diagnosis or consensus diagnosis • Most relevant to real-world diagnosis at presentation • Most feasible for retrospective datasets Histopathology • Traditional tissue-based reference standard • High specificity when tissue is available Genetic profiling • Adds biologic precision • Provides objective, stable labels
Management decision	Prospective clinical trial • Captures outcomes associated with specific presentations and management decisions • Particularly relevant for treat-versus-observe decisions in borderline or genetically low-risk lesions • Randomized trials are ideal when feasible across tumor sizes, features, and genetic profiles	Consensus recommendation • Reflects expert intended management • Reduces single-clinician noise • Can be difficult to reach agreement Observed treatment decision • Readily available in retrospective datasets • Incorporates real-world constraints and patient preference • Does not consider treatment plans not chosen due to these constraints
Radiation Treatment Planning	Expert manual annotation • Supports accurate tumor segmentation and dosimetric planning • Directly reflects clinician input required for treatment planning	Historic clinician-approved treatment plans • Scalable for supervised learning from prior care • May capture institutional planning preferences rather than optimal plans
Long-term disease- specific outcome risk	Prospective monitoring • Directly reflects clinically meaningful endpoints • Enables time-to-metastasis modeling	LUMPO staging • Integrates multiple clinicopathologic factors • Widely understood across specialties • Useful for population-level risk stratification but less individualized
Patient counseling	Genetic profiling • Provides individualized metastatic risk information • Often the most actionable information for prognosis-focused counseling	Clinical and size-based prognostic factors • Non-invasive and broadly available • Less biologically specific but often sufficient for initial counseling Histopathology • Can provide additional prognostic information • Greater availability in some countries

## Data Availability

No new data were created or analyzed in this study.

## Abbreviations

The following abbreviations are used in this manuscript:

UM	Uveal melanoma
AI	Artificial intelligence
ML	Machine learning
OCT	Optical coherence tomography
COMS	Collaborative Ocular Melanoma Study
AJCC	The American Joint Committee on Cancer
LUMPO	Liverpool Uveal Melanoma Prognosticator Online
IMCT	Indeterminate melanocytic choroidal tumors
GEP	Gene expression profiling
MOLES	Mushroom shape, Orange pigment, Large size, Enlarging tumor, Subretinal fluid
TFSOM	Thickness, subretinal Fluid, Symptoms, Orange pigment, Margin
NCCN	National Comprehensive Cancer Network
RCT	Randomized controlled trial
